# Impact of religion and spirituality on the incidence of depression and mental health among young adults in the Czech Republic

**DOI:** 10.3389/fpsyg.2024.1423730

**Published:** 2024-08-29

**Authors:** Tibor A. Brečka, Radek Ptáček, Ivan Sebalo, Martin Anders, Martina Sebalo Vňuková

**Affiliations:** Department of Psychiatry, 1st Faculty of Medicine, Charles University and General University Hospital Prague, Prague, Czechia

**Keywords:** religion, spirituality, mental health, young adults, psychology

## Abstract

**Introduction:**

The issue of the impact of religion and spirituality on mental health is a phenomenon which has recently become increasingly more accentuated. Despite the attention given to the topic, many questions still remain as to whether and how religion and spirituality affect a person’s mental wellbeing. In the text below, we have focused on examining the relationship between religion and spirituality and mental health among young adults in the Czech Republic. Research also explored the idea that forgiveness can be viewed as a component of religion or spirituality.

**Materials and methods:**

The research project was executed in close cooperation with STEM/MARK, a renowned data collection agency. The methodological framework was constructed with a dual focus: leveraging standardized questionnaires to ensure data reliability and comparability while also incorporating tailored questions that delve into the participants’ socioeconomic status (SES) and background details. The study unfolded across four online sessions, a format chosen for its convenience and effectiveness in facilitating participant engagement while accommodating our respondents’ diverse schedules. The total sample approached comprised of 270 young adults that expressed certain form of religiosity.

**Results:**

Forgiveness and the depth of one’s personal religious or spiritual history emerged as the most influential factors. Forgiveness was significantly associated with an increase in self-blame (positively), and decrease in refocusing, planning (both negatively), and putting things into perspective (negatively) (Beta = 0.25, Beta = −0.06, and Beta = −0.16, respectively). In contrast, a deeper personal religious history was positively associated with self-blame, rumination, and refocusing (Beta = 0.22, Beta = 0.13, and Beta = 0.15, respectively).

**Conclusion:**

The finding that forgiveness may be a risk factor associated with regularly elevated depressive symptoms, stress, and maladaptive coping strategies such as self-blame and ruminating over problems, while negatively affecting physical, psychological, and environmental quality of life, clearly points to the need to examine the inner aspects of individual religions and spiritualities. These findings suggest that religious and spiritual beliefs may play a key role in how people experience and manage the emotional burdens and difficulties of life.

## Introduction

The impact of religion and spirituality on mental health has garnered increased attention in recent years. However, many questions remain about how and to what extent these factors influence mental wellbeing. Given the broad and complex nature of ‘mental health’ (Brečka et al., 2023), this study will focus specifically on the relationship between religion, spirituality, and depressive symptoms.

Like the term ‘mental health’, ‘religion’ and ‘spirituality’ are fairly complex, and we have already examined them in depth elsewhere (Brečka et al., 2022). Hence, we will provide only a brief outline of our view of this issue here. There are numerous definitions of religion and spirituality. In extremely simple terms, the difference may be seen in that religion refers to institutionalized, tradition-focused religious conviction and practices, whereas spirituality refers to non-institutionalized religion and customs practices focused on tradition (Říčan, 2007). Yet even this division is flawed. We find that the best way to illustrate the correlation between these two terms is to depict is as a Mobius strip. A Mobius strip is a surface that has only one side and one edge. Now, is the sole surface of a Mobius strip its face or its reverse? It is impossible to determine, just as it is impossible to determine where spirituality ends and religion begins (Brečka et al., 2022).

At first glance, it may seem that the connection between religion/spirituality and depression has been clearly established. A systematic review of quantitative research up to 2010 (both observational and experimental work) reported that 444 studies had examined the relationship between religion/spirituality and depression (Koenig et al., 2012). Of those studies, 272 (61%) reported that religious involvement was associated with less depression or faster recovery from depression, or that religious interventions significantly reduced depressive symptoms (Koenig et al., 2020). Another systematic review of scientific papers on this topic over the past 5 years was conducted in 2021 with a similar outcome (Papaleontiou-Louca, 2021). A positive correlation between religion, spirituality and a lower degree of depressive symptomatology is mentioned, among others, by Garduño-Ortega et al. (2021), De Berardis et al. (2020), Chen et al. (2021), Milstein et al. (2020), and Sarizadeh et al. (2020). The same positive correlation was identified in a number of studies examining the impacts of the COVID-19 pandemic (Kimhi et al., 2021; Pirutinsky et al., 2020; Moura et al., 2022; Mahamid and Bdier, 2021).

Nevertheless, questions continue to arise as to whether the connection between religion, spirituality and the symptoms of depressive illness is indeed so clear, and what other factors contribute to this correlation. These relationships are understudied in young people. For instance, Fradelos et al. states that “According to [their] results although religious practices can be a protective factor for both depression and anxiety, religious beliefs and experiences can increase the levels of depression and anxiety as well” (Fradelos et al., 2020). What are the factors that can cause religion and spirituality to influence the symptoms of depressive illness, either in a positive or negative manner?

The first factor may be the actual type of religion and spirituality. As stated by Schnittker is his study (Schnittker, 2020). Similar results in the Islamic religion were reported in the study by Bakır et al. (2021).

Bakir’s study, however, raises the issue of another factor closely associated with the examined issue, that being ethnicity. Ethnicity in itself has a major impact on an individual’s religious/ spiritual orientation. This is confirmed in the study by Tan et al. “Data of 7,068 participants (4,418 Malays, 2080 Chinese and 570 Indians) aged ≥55 years that were collected as part of the community health survey conducted in 2013 in the South East Asia Community Observatory (SEACO) were analyzed using bivariate and multiple regressions. Analyses were stratified by ethnicity. The importance of having an enriched religious/spiritual life was associated with higher scores of depression, anxiety and stress among Chinese and higher score of depression among Malays, while belief in a higher power was associated with better mental health among Malays, Chinese and Indians” (Tan et al., 2021).

Ethnicity is also accentuated in the study by Haney and Rollock (2020), which mentions yet another interesting factor, that being gender. The study examined several domains of religion as contributors to mental health, distinct from personality. A sample of emerging adults (*n* = 509) reported on these constructs along with measures of depression, anxiety, aggression, satisfaction with life, and flourishing. As hypothesized, hierarchical multiple regressions indicated that higher levels of religiosity were associated with better mental health outcomes above and beyond demographic characteristics and personality, and religious doubt was associated with poorer outcomes. Religiosity also differed as a function of gender and ethnicity. Differences between men and women are likewise reported in the study by Fukai et al. (2020).

Gender differences are pointed out by Kent, who adds another factor, namely age and changes in experience religion/spirituality with respect to age (Kent, 2020). Age and changes depending on age and aging are also reported in the study by Anderson et al. (2020).

In addition to the age factor, Unpenieks and Thomas also addressed the impact of education. “Using the life course perspective, [they] assess the ‘resources’ and ‘risks’ to mental health associated with transitions in religious attendance between early life and midlife and how this process may be influenced by education. Drawing on over 35 years of prospective panel data from the National Longitudinal Study of Youth, baseline models suggest that stable, frequent attendance accumulated between adolescence to midlife and increases to frequent attendance by adulthood are associated with the lowest depression relative to consistent nonattenders. Individuals who declined in their religious participation report higher depression. Education conditioned this association, whereby declines in religious participation negatively impacted the health of those without a college degree more strongly and increases benefitted the well-educated to a greater extent. [They] combine insights from the life course perspective and work on social stratification and religiosity to interpret [their] results and offer directives for future research.” (Upenieks and Thomas, 2021).

Finally, but equally worth mentioning, is the issue of personal characteristics and religion/spirituality, meaning whether personality affects the choice of spirituality and religion we encounter among converts. Shestakov and Kňažek offer an interesting elaboration on this theme, in that “based on the results of research, 86 of the total number of respondents displayed accentuated traits of certain characteristics, or a tendency toward character accentuations. In this respect, it should be noted that the obtained results allow [the researchers] to assume that the choice of a certain religious system may correlate with an individual’s personality traits” (Shestakov and Kňažek, 2021).

Nevertheless, what we consider to be the most important factor, like Shabit et al., is the question of active fatalism. [They] found that while classic fatalism was significantly and positively associated with depression and negative coping, active fatalism was positively correlated with positive coping skills, and negatively correlated with depression and external locus of control (Fatalism is the belief that all events are predetermined and cannot be influenced by human will. Classic fatalism specifically refers to the belief that fate is fixed, and individuals have no control over their lives or the events that happen to them). The significant positive correlation of positive coping and negative correlations of depression and external locus of control with active fatalism offer evidence in support of the notion that this form of fatalism may in fact be associated with protective mechanisms against depression. [The researchers] defined active fatalism as the belief in a predestined personal and global future, combined with the belief that one must do their part to bring this predestined future into fruition.” (Shahid et al., 2020).

According to recent studies, spirituality, as opposed to institutionalized religion, has a specific impact on mental health and can act as a protective factor against depression. Research shows that regular spiritual practices such as meditation and prayer can increase the thickness of the cortical area in the prefrontal region of the brain, which can help alleviate depression symptoms and reduce feelings of hopelessness (Miller, 2019; Jackson, 2019). Studies focused on interventions to improve spiritual well-being have shown positive results in reducing depressive and anxiety symptoms. For example, a seven-week program of Hatha yoga and meditation led to a significant reduction in anxiety and depression among university students (Rickhi et al., 2011; BMC Psychiatry, 2021). Similar results were observed in interventions based on Buddhist meditation practices (BMC Psychiatry, 2021). Research also indicates that spirituality can serve as a shield against stress and provide new ways of coping with depression. By focusing the mind on a higher power or seeking meaning through spiritual practices, individuals can change their perspective on life and improve their mental health (Jackson, 2019; BMC Psychiatry, 2021).

In this section, we initially focus on age differences, gender, and fatalism to provide a comprehensive context for our study. Although these factors are not extensively discussed later, they offer important insights into the demographic and psychological background of our participants. Nevertheless, we recognize the significance of forgiveness as ‘a central aspect of some religious doctrines’. Therefore, we have incorporated a detailed analysis of forgiveness and its impact on depressive symptoms in the subsequent sections to address its crucial role as highlighted in our findings.

### Impact of forgiveness on depressive symptoms

Recent studies have demonstrated that forgiveness has a significant impact on mental health, particularly in reducing depressive symptoms. Research shows that forgiveness of others and self-forgiveness are both inversely related to depressive symptoms. For instance, higher levels of forgiveness are associated with lower levels of psychological distress and higher levels of psychosocial well-being (Harris and Thoresen, 2005; Hoyt et al., 2005). A longitudinal study on female nurses found that those who practiced forgiveness reported higher levels of positive affect and lower levels of psychological distress over time. This suggests that forgiveness not only has immediate mental health benefits but also contributes to long-term psychological well-being (BMC Psychology, 2021). Forgiveness may impact depressive symptoms through several mechanisms. It can reduce rumination and negative thinking, which are significant contributors to depression. Moreover, forgiveness is associated with improved interpersonal relationships, which can provide emotional support and reduce feelings of isolation and loneliness (Lawler-Row et al., 2008; McCullough et al., 2007). Interventions that promote forgiveness have shown to be effective in reducing depressive symptoms. These interventions often include cognitive-behavioral techniques, mindfulness, and spiritual practices that help individuals process and release feelings of anger and resentment. For example, a study found that a forgiveness intervention led to significant reductions in depressive symptoms and increased psychological resilience among participants (Karremans and van Lange, 2008).

In the study, we have identified several key areas that are relevant for examining the impact of R/S on mental health:

1. Types of Religiosity and Spirituality: Subsequent analyses will focus on various types of religiosity (e.g., institutionalized religion vs. personal spirituality) and their specific effects on mental health. This section will include analyses examining how different forms of R/S may have protective or risk effects on depression symptoms (see “Material and Methods” section in the manuscript).
2. Forgiveness: As stated in the introductory section, forgiveness is a key aspect of some religious doctrines. Subsequent analyses will focus on the quantitative measurement of the impact of forgiveness on depressive symptoms and stress (see “Results” section in the manuscript). Research shows that forgiveness can have a significant impact on reducing negative emotions and increasing overall psychological well-being. For example, our analysis revealed that forgiveness is significantly associated with an increase in self-blame (positively) and a decrease in positive refocusing and planning (both negatively) (see Table 1). These patterns indicate that aspects of religiosity, such as forgiveness, may primarily be linked to maladaptive coping strategies, such as self-blame and rumination, and may negatively affect more adaptive coping strategies, like refocusing (see Table 1).
3. Demographic Factors: Although age and gender are not the main factors of this study, they are important for understanding the broader context. Analyses show that different demographic groups may have varying experiences and approaches to R/S, which can affect their mental health. For example, our analysis shows that demographic factors such as age and gender may play a role in individual differences in responses to religious and spiritual practices (see Table 2).
4. Fatalism: This area, although not the main focus, was mentioned for its theoretical relevance. Fatalistic beliefs can influence how individuals interpret and manage their life situations, which has a direct impact on their mental health (Shahid et al., 2020). Our analysis shows that some parts of fatalistic approach may be associated with negative coping strategies and higher levels of stress (see Table 3).

**Table 1:** Associations between religiousness domains on coping strategies (*n* = 270).

	Self-Blame	Acceptance	Rumination	Positive refocusing	Refocus planning	Positive reappraisal	Putting into perspective	Catastrophizing	Blaming others
	Estimate, (95% CI)	Estimate, (95% CI)	Estimate, (95% CI)	Estimate, (95% CI)	Estimate, (95% CI)	Estimate, (95% CI)	Estimate, (95% CI)	Estimate, (95% CI)	Estimate, (95% CI)
	(1)	(2)	(3)	(4)	(5)	(6)	(7)	(8)	(9)
(Constant)	7.26^***^ (4.50, 9.93)	11.60^***^ (9.19, 14.10)	11.71^***^ (9.00, 14.44)	12.89^***^ (10.35, 15.48)	12.30^***^ (9.77, 14.89)	13.19^***^ (10.46, 16.12)	13.02^***^ (10.12, 15.85)	8.94^***^ (6.45, 11.40)	10.53^***^ (8.33, 12.76)
Daily spiritual experiences	−0.06 (−0.15, 0.05)	−0.06 (−0.15, 0.04)	−0.07 (−0.17, 0.04)	−0.06 (−0.17, 0.03)	−0.08^*^ (−0.17, 0.01)	−0.09 (−0.19, 0.03)	−0.08 (−0.18, 0.05)	−0.04 (−0.12, 0.06)	−0.09^*^ (−0.19, 0.01)
Values	−0.16 (−0.61, 0.24)	0.08 (−0.37, 0.49)	−0.06 (−0.52, 0.34)	0.07 (−0.41, 0.51)	−0.02 (−0.46, 0.35)	−0.22 (−0.74, 0.23)	−0.06 (−0.57, 0.41)	0.004 (−0.39, 0.43)	0.14 (−0.22, 0.46)
Forgiveness	0.43^***^ (0.19, 0.67)	−0.11 (−0.35, 0.15)	0.17 (−0.08, 0.43)	−0.40^***^ (−0.64, −0.12)	−0.11 (−0.35, 0.15)	−0.21 (−0.47, 0.08)	−0.31^*^ (−0.59, 0.004)	0.18 (−0.06, 0.42)	0.08 (−0.13, 0.30)
Private religious practice	0.03 (−0.09, 0.16)	0.10 (−0.02, 0.23)	0.07 (−0.07, 0.20)	0.02 (−0.10, 0.15)	0.09 (−0.03, 0.22)	0.05 (−0.07, 0.19)	0.06 (−0.08, 0.22)	−0.04 (−0.19, 0.11)	−0.05 (−0.17, 0.10)
Religious coping	−0.02 (−0.15, 0.11)	−0.14^*^ (−0.26, −0.001)	−0.12 (−0.25, 0.02)	−0.05 (−0.16, 0.08)	−0.09 (−0.21, 0.04)	−0.10 (−0.23, 0.05)	−0.05 (−0.19, 0.11)	−0.06 (−0.19, 0.06)	−0.05 (−0.16, 0.07)
Personal religious history	0.90^***^ (0.42, 1.39)	0.34 (−0.12, 0.80)	0.53^*^ (0.04, 1.03)	0.05 (−0.46, 0.59)	0.58^*^ (0.12, 1.05)	0.36 (−0.24, 0.96)	0.30 (−0.27, 0.91)	0.18 (−0.32, 0.70)	−0.26 (−0.73, 0.21)
Organizational religiousness	0.09 (−0.16, 0.34)	0.01 (−0.25, 0.25)	0.12 (−0.13, 0.36)	0.13 (−0.13, 0.37)	0.12 (−0.14, 0.34)	0.26 (−0.01, 0.50)	0.18 (−0.12, 0.44)	0.02 (−0.23, 0.30)	0.07 (−0.18, 0.31)
Self-ranking as religious	0.10 (−0.46, 0.59)	0.37 (−0.13, 0.80)	−0.10 (−0.68, 0.42)	0.21 (−0.28, 0.65)	0.05 (−0.47, 0.52)	0.25 (−0.28, 0.71)	0.16 (−0.37, 0.61)	0.22 (−0.39, 0.75)	0.07 (−0.47, 0.56)
Observations	270	270	270	270	270	270	270	270	270
R^2^	0.10	0.06	0.08	0.09	0.10	0.09	0.07	0.02	0.04
Adjusted R^2^	0.07	0.03	0.06	0.06	0.07	0.06	0.04	−0.01	0.02
Residual Std. Error (df = 261)	3.50	3.48	3.54	3.36	3.36	3.93	3.91	3.55	3.12
F Statistic (df = 8; 261)	3.69^***^	1.95	2.96^**^	3.08^**^	3.43^***^	3.15^**^	2.43^*^	0.73	1.52

*p < 0.05; **p < 0.01; ***p < 0.001.

**Table 2:** Descriptive statistics of the sample.

	Consider themselves believers (*n* = 270)	Do not consider themselves believers (*n* = 659)	Do not know (101)	Total (*n* = 1,030)
Sex	*N* (%)	*N* (%)	*N* (%)	*N* (%)
Male	131 (25.84)	331 (65.29)	45 (8.88)	507 (100.00)
Female	138 (26.54)	326 (62.69)	56 (10.77)	520 (100.00)
Missing	1 (33.33)	2 (66.67)	0 (0.00)	3 (100.00)
Do you consider spirituality a part of your life?				
Yes	172 (56.77)	94 (31.02)	37 (12.21)	303 (100.00)
No	98 (13.48)	565 (77.72)	64 (8.80)	727 (100.00)
Do you consider religion a part of your life?				
Yes	146 (86.90)	15 (8.93)	7 (4.17)	168 (100.00)
No	124 (14.39)	644 (74.71)	94 (10.90)	862 (100.00)
Nature is a very important and integral part of my religious/spiritual orientation				
I do not agree	35 (10.20)	272 (79.30)	36 (10.50)	343 (100.00)
I definitely disagree	3 (2.33)	124 (96.12)	2 (1.55)	129 (100.00)
I definitely agree	97 (59.15)	55 (33.54)	12 (7.32)	164 (100.00)
I agree	135 (34.26)	208 (52.79)	51 (12.94)	394 (100.00)
Is your religion/spirituality more of a personal or group/public affair?				
Totally group/public, sharing and celebrating together is an absolute staple	13 (100.00)	0 (0.00)	0 (0.00)	13 (100.00)
Totally private, I do not share it with anyone else	101 (49.75)	74 (36.45)	28 (13.79)	203 (100.00)
Rather group/public, I share with a large number of other members	23 (88.46)	3 (11.54)	0 (0.00)	26 (100.00)
Rather private, I only share with a small number of members	79 (68.10)	26 (22.41)	11 (9.48)	116 (100.00)
Missing	54 (8.04)	556 (82.74)	62 (9.23)	672 (100.00)
How spiritual would you describe yourself?				
Strongly spiritual	31 (100.00)	0 (0.00)	0 (0.00)	31 (100.00)
Mildly spiritual	117 (100.00)	0 (0.00)	0 (0.00)	117 (100.00)
Moderately spiritual	83 (100.00)	0 (0.00)	0 (0.00)	83 (100.00)
Not spiritual at all	39 (100.00)	0 (0.00)	0 (0.00)	39 (100.00)
Missing	0 (0.00)	659 (86.71)	101 (13.29)	760 (100.00)
Beck Anxiety Inventory				
Mean (Sd)	12.95 (10.89)	11.61 (10.88)	12.94 (11.43)	12.09 (10.95)
Range	0–59	0–54	0–46	0–59
Beck Depression Inventory				
Mean (Sd)	14.15 (10.83)	13.11 (10.85)	14.34 (11.85)	13.5 (10.95)
Range	0–55	0–53	0–57	0–57
Expressions of spirituality inventory cognitive orientation toward spirituality dimension				
Mean (Sd)	13.06 (5.22)	6.02 (4.89)	10.03 (4.96)	8.26 (5.86)
Range	0–24	0–24	0–24	0–24
Expressions of spirituality inventory experiential/phenomenological dimension				
Mean (Sd)	9.57 (5.03)	5.14 (4.74)	8.15 (5.05)	6.6 (5.23)
Range	0–21	0–24	0–19	0–24
Expressions of spirituality inventory existential well-being dimension				
Mean (Sd)	14.06 (4.58)	15.11 (4.77)	13.56 (4.4)	14.68 (4.72)
Range	0–24	0–24	0–24	0–24
Expressions of spirituality inventory paranormal beliefs dimension				
Mean (Sd)	10.65 (3.9)	7.79 (4.11)	10.14 (3.56)	8.77 (4.21)
Range	0–20	0–24	0–19	0–24
Expressions of spirituality inventory religiousness dimension				
Mean (Sd)	11.7 (4.94)	3.81 (4.18)	6.87 (4.02)	6.18 (5.55)
Range	0–24	0–24	0–15	0–24
Symptom checklist aggressivity/hostility				
Mean (Sd)	0.69 (0.76)	0.61 (0.76)	0.77 (0.87)	0.65 (0.77)
Range	0–3.5	0–4	0–4	0–4
Symptom checklist anxiety				
Mean (Sd)	0.75 (0.78)	0.6 (0.7)	0.78 (0.86)	0.66 (0.74)
Range	0–4	0–4	0–4	0–4
Symptom checklist depression				
Mean (Sd)	0.98 (0.88)	0.88 (0.82)	1.04 (0.93)	0.92 (0.85)
Range	0–3.62	0–3.92	0–3.62	0–3.92
Symptom checklist paranoid ideation				
Mean (Sd)	0.93 (0.9)	0.73 (0.78)	0.88 (0.86)	0.8 (0.82)
Range	0–3.67	0–3.83	0–4	0–4
Symptom checklist phobic-anxiety				
Mean (Sd)	0.47 (0.64)	0.44 (0.62)	0.48 (0.64)	0.45 (0.63)
Range	0–3.86	0–3	0–3.29	0–3.86
Symptom checklist psychoticism				
Mean (Sd)	0.56 (0.7)	0.47 (0.6)	0.54 (0.66)	0.5 (0.63)
Range	0–3.3	0–3.3	0–3.6	0–3.6
Symptom checklist somatization				
Mean (Sd)	0.6 (0.67)	0.53 (0.63)	0.62 (0.73)	0.56 (0.65)
Range	0–3.08	0–3.75	0–4	0–4
Symptom checklist interpersonal sensibility				
Mean (Sd)	0.94 (0.88)	0.77 (0.85)	0.95 (0.93)	0.83 (0.87)
Range	0–3.78	0–4	0–4	0–4
Symptom checklist obsessive-compulsive				
Mean (Sd)	0.92 (0.84)	0.77 (0.75)	0.92 (0.87)	0.82 (0.79)
Range	0–3.9	0–3.7	0–4	0–4
Symptom checklist GSI				
Mean (Sd)	0.77 (0.7)	0.66 (0.64)	0.78 (0.74)	0.7 (0.67)
Range	0–3.53	0–3.58	0–3.79	0–3.79
Symptom checklist PST				
Mean (Sd)	38.17 (25.51)	33.98 (25.71)	37.84 (25.79)	35.46 (25.71)
Range	0–90	0–90	0–90	0–90
Symptom checklist PSDI				
Mean (Sd)	1.59 (0.52)	1.53 (0.5)	1.6 (0.59)	1.55 (0.51)
Range	1.3.1961	1.3.1958	1.3.1988	1.3.1988
World Health Organization quality of life scale overall quality of life				
Mean (Sd)	7.42 (1.67)	7.42 (1.58)	7.26 (1.56)	7.41 (1.6)
Range	3–10	2–10	3–10	2–10
World Health Organization quality of life scale physical health				
Mean (Sd)	21.39 (3.3)	21.66 (3.25)	21.21 (3.45)	21.54 (3.28)
Range	12–35	13–31	13–33	12–35
World Health Organization quality of life scale psychological healthy				
Mean (Sd)	19.48 (3.51)	19.3 (3.57)	18.82 (3.75)	19.3 (3.57)
Range	8–27	7–28	9–27	7–28
World Health Organization quality of life scale social relations				
Mean (Sd)	10.49 (2.66)	10.6 (2.57)	10.02 (2.69)	10.51 (2.61)
Range	3–15	3–15	3–15	3–15
World Health Organization quality of life scale environmental health				
Mean (Sd)	28.6 (5.29)	29.29 (5.02)	27.75 (5.2)	28.96 (5.13)
Range	12–40	11–40	17–40	11–40

**Table 3:** Associations between religiousness domains on mental health (*n* = 270).

	BDI (*n* = 270)	BAI (*n* = 270)	Stress (*n* = 270)
	Estimate, (95% CI)	Estimate, (95% CI)	Estimate, (95% CI)
	(1)	(2)	(3)
(Constant)	8.46^*^ (0.72, 16.33)	19.34^***^ (10.79, 27.89)	17.07^***^ (12.92, 21.15)
Daily Spiritual Experiences	−0.01 (−0.33, 0.35)	−0.07 (−0.37, 0.25)	0.04 (−0.11, 0.18)
Values	−0.61 (−1.90, 0.74)	−0.34 (−1.58, 0.84)	−0.23 (−0.93, 0.49)
Forgiveness	1.05^**^ (0.32, 1.72)	0.34 (−0.40, 1.05)	0.81^***^ (0.41, 1.19)
Private Religious Practice	−0.02 (−0.43, 0.38)	0.05 (−0.34, 0.44)	0.06 (−0.12, 0.24)
Religious Coping	0.08 (−0.36, 0.48)	0.09 (−0.31, 0.46)	−0.13 (−0.37, 0.09)
Personal Religious History	1.62^*^ (0.04, 3.13)	0.80 (−0.91, 2.48)	0.58 (−0.31, 1.40)
Organizational Religiousness	−0.26 (−1.04, 0.58)	−0.47 (−1.30, 0.40)	−0.41 (−0.78, −0.01)
Self-Ranking as Religious	0.22 (−1.11, 1.51)	−0.88 (−2.32, 0.60)	0.16 (−0.61, 0.92)
Observations	270	270	270
R^2^	0.05	0.04	0.07
Adjusted R^2^	0.02	0.01	0.04
Residual Std. Error (df = 261)	10.74	10.84	6.23
F Statistic (df = 8; 261)	1.59	1.30	2.39^*^

*p < 0.05; **p < 0.01; ***p < 0.001.

The current research aims to contribute new insights to the field of religiosity and spirituality (R/S) and its impact on mental health, specifically focusing on depressive symptoms. The goals and interests of this study are to: Differentiate Types of R/S: Investigate how different types of religiosity and spirituality (e.g., institutionalized religion vs. personal spirituality) uniquely influence depressive symptoms. This study seeks to provide a detailed understanding of the protective or risk factors associated with each type. Explore the role of forgiveness, which is described as a central aspect of some religious doctrines, and its impact on mental health. The study will analyze how forgiveness affects negative emotions and contributes to overall psychological well-being. Examine how demographic factors such as age and gender relate to R/S and its effects on mental health. By understanding these variations, the study aims to offer a more comprehensive view of how different populations experience and benefit from R/S practices. By addressing these objectives, this research aims to fill gaps in the existing literature, offering a comprehensive analysis of the various aspects of R/S and their contributions to mental health, particularly in the context of depressive symptoms.

## Materials and methods

The research project was executed in close cooperation with STEM/MARK, data collection agency. Our methodological framework was constructed with a dual focus: leveraging standardized questionnaires to ensure data reliability and comparability while also incorporating tailored questions that delve into the participants’ socioeconomic status (SES) and background details.

Recruitment of participants was strategically managed through the agency’s European national panel, which is known for its diverse and representative nature. This selection process was critical in ensuring that our participant pool mirrored the demographic characteristics of young Czech adults, with particular attention to gender, education level, SES, and other pivotal indicators.

The Brief Multidimensional Measure of Religiousness/Spirituality (BMMRS) is a tool developed to assess various dimensions of religiosity and spirituality. Originally created for use in social, behavioral, and health sciences, BMMRS includes several scales such as Daily Spiritual Experience, Religious Coping, and Values, allowing for a comprehensive assessment of the spiritual and religious aspects of an individual’s life. The psychometric properties of BMMRS have been evaluated in various studies, demonstrating the tool’s reliability and validity. For instance, studies indicate that BMMRS exhibits good internal consistency, with Cronbach’s alpha for different scales typically exceeding 0.80, suggesting high internal consistency (Fetzer Institute, National Institute on Aging Working Group, 1999). The validity of the tool has been confirmed through convergent and discriminant validity in relation to other measures of religiosity and spirituality, supporting its use in various research contexts.

The Existential Spirituality Inventory-Revised (ESI-R) is a tool designed to assess existential spirituality, which includes aspects such as personal meaning, the sense of purpose in life, and deeper existential questions. This instrument is particularly useful in evaluating how individuals find meaning and purpose beyond traditional religious frameworks. The psychometric properties of ESI-R have been evaluated in several studies, demonstrating its reliability and validity. Research indicates that ESI-R shows strong internal consistency, with Cronbach’s alpha values for its subscales generally exceeding 0.80, suggesting high reliability (MacDonald, 2000). The tool’s validity has been supported through various forms of validation, including convergent validity with other measures of spirituality and existential well-being, and discriminant validity, indicating that ESI-R effectively measures distinct aspects of existential spirituality (MacDonald, 2000). Additionally, factor analysis has confirmed the multidimensional structure of ESI-R, validating its use in capturing the complex nature of existential spirituality (MacDonald, 2000). These psychometric properties make ESI-R a robust instrument for research in existential spirituality and its impact on mental health and well-being.

The study unfolded across four online questionnaire sessions spanning throughout approximately one hour, a format chosen for its convenience and effectiveness in facilitating participant engagement while accommodating our respondents’ diverse schedules. The exclusion criteria included severe form of mental disorder that impacts cognitive abilities as well as any form of legal incompetence that would prevent the participant to sign informed consent for themselves. Thus inclusion criteria were healthy young adults aged 18–35. For this sub-study only questionnaires regarding religiousness and spirituality were analyzed.

### Analysis plan

The data were analyzed using R software, with multiple linear regression as the primary analysis method. To ensure robustness against violations of normality and to protect against the effects of repeated testing, 95% Confidence Intervals based on 5,000 bootstraps were used to verify the *p*-values. Additionally, the potential for multicollinearity was assessed using the Variance Inflation Factor (VIF) for the subscales of one scale used in most models. In no case did the VIF exceed 5. Afterwards a parallel multiple mediation analysis was performed.

## Results

The total sample comprised 1,030 young adults with an average age of 24.52 (SD = 3.98). It featured an almost equal distribution of males (49%) and females (50%), with three participants identifying as nonbinary. Most of the sample did not identify with any religion (64%), while 26% considered themselves believers regardless of affiliation with a specific religion and 10% were undecided. Table 2 provides a detailed description of the sample.

The initial phase of the analysis focused on assessing the impact of various dimensions of religiosity and spirituliaty, assessed via Brief Multidimensional Measure of Religiousness/Spirituality (BMMRS) on mental health outcomes (Fetzer Institute, National Institute on Aging Working Group, 1999). Since BMMRS was administered only to those who consider themselves to be believers, subsequent models were constructed for 270 participants. Due to this sample size limitation, all included variables are present in the tables presenting results of the respective analyses. The regression model that predicted depressive symptoms using religiosity factors was not a good fit. Notably, forgiveness (Beta = 0.21) and spiritual history (Beta = 0.13) were positively associated with depressive symptom scores on the Beck Depression Inventory II (BDI), as detailed in Table 3. This result implies that higher levels of forgiveness and a more substantial spiritual history may correlate with an increase in depressive symptoms.

Subsequent analysis with anxiety symptoms as the dependent variable revealed that the model was a poor fit and no predictors were significant. This indicates a lack of relationship between religiosity and anxiety symptoms.

In contrast, when exploring the model with self-reported stress levels as the outcome, the fit was good, albeit explaining only a modest 4% of the variance. The only significant predictor in this model was forgiveness (Beta = 0.27), indicating that individuals who frequently engage in forgiveness, as dictated by their religious practices, tend to report higher stress levels on average.

In the next phase of analysis, we examined the influence of religiosity on mental health symptomatology. According to the regression models detailed in Table 4, none exhibited a satisfactory fit to the data, indicating limitations in their explanatory power for the observed mental health outcomes. However, forgiveness emerged as a significant positive predictor across nearly all domains of the Symptom Checklist-90-Revised (SCL-90-R), notably within the realms of Hostility (Beta = 0.15), Depression (Beta = 0.15), Paranoid Ideation (Beta = 0.16), Psychoticism (Beta = 0.16), and Interpersonal Sensitivity (Beta = 0.17). These findings suggest that incorporating forgiveness as an element of one’s religious practice may be associated with exacerbations in various facets of mental health.

**Table 4:** Associations between religiousness domains on SCL subscales (*n* = 270).

	Hostility	Anxiety	Depression	Paranoid Ideation	Phobic Anxiety	Psychoticism	Somatization	Interpersonal Sensitivity	Obsessive-Compulsive
	Estimate, (95% CI)	Estimate, (95% CI)	Estimate, (95% CI)	Estimate, (95% CI)	Estimate, (95% CI)	Estimate, (95% CI)	Estimate, (95% CI)	Estimate, (95% CI)	Estimate, (95% CI)
	(1)	(2)	(3)	(4)	(5)	(6)	(7)	(8)	(9)
(Constant)	0.88^**^ (0.27, 1.49)	1.01^***^ (0.44, 1.59)	0.89^**^ (0.27, 1.52)	1.09^**^ (0.44, 1.78)	0.90^***^ (0.40, 1.42)	0.83^**^ (0.32, 1.39)	1.02^***^ (0.49, 1.55)	0.96^**^ (0.31, 1.62)	1.03^**^ (0.44, 1.65)
Daily Spiritual Experiences	−0.01 (−0.03, 0.01)	−0.01 (−0.03, 0.02)	−0.004 (−0.03, 0.03)	−0.02^*^ (−0.05, 0.01)	−0.01 (−0.03, 0.01)	−0.01 (−0.03, 0.01)	−0.01 (−0.02, 0.01)	−0.01 (−0.04, 0.02)	−0.01 (−0.03, 0.02)
Values	−0.005 (−0.09, 0.09)	0.03 (−0.05, 0.12)	0.06 (−0.05, 0.16)	0.002 (−0.10, 0.11)	−0.02 (−0.09, 0.05)	0.01 (−0.06, 0.08)	0.02 (−0.05, 0.10)	−0.02 (−0.13, 0.09)	0.02 (−0.08, 0.11)
Forgiveness	0.05^*^ (−0.002, 0.10)	0.04 (−0.01, 0.09)	0.06^*^ (0.01, 0.12)	0.07^*^ (0.01, 0.13)	0.04 (−0.01, 0.08)	0.05^*^ (0.01, 0.10)	0.02 (−0.02, 0.06)	0.07^*^ (0.01, 0.13)	0.05 (−0.01, 0.11)
Private Religious Practice	0.001 (−0.03, 0.03)	−0.01 (−0.04, 0.02)	0.01 (−0.02, 0.04)	−0.001 (−0.03, 0.03)	−0.01 (−0.04, 0.02)	−0.01 (−0.04, 0.01)	−0.01 (−0.04, 0.02)	0.005 (−0.02, 0.03)	0.003 (−0.03, 0.03)
Religious Coping	0.002 (−0.03, 0.03)	−0.001 (−0.03, 0.02)	−0.01 (−0.05, 0.02)	−0.002 (−0.04, 0.03)	0.02 (−0.01, 0.04)	0.001 (−0.03, 0.03)	0.004 (−0.02, 0.03)	0.01 (−0.03, 0.04)	−0.002 (−0.04, 0.03)
Personal Religious History	0.06 (−0.06, 0.19)	0.07 (−0.05, 0.19)	0.09 (−0.04, 0.22)	0.11 (−0.04, 0.24)	−0.01 (−0.12, 0.10)	0.03 (−0.08, 0.14)	0.01 (−0.10, 0.11)	0.11 (−0.03, 0.23)	0.08 (−0.04, 0.21)
Organizational Religiousness	−0.03 (−0.09, 0.03)	−0.02 (−0.07, 0.04)	−0.03 (−0.09, 0.03)	−0.02 (−0.09, 0.05)	−0.02 (−0.07, 0.03)	−0.01 (−0.06, 0.05)	−0.02 (−0.07, 0.04)	−0.04 (−0.10, 0.03)	−0.03 (−0.09, 0.03)
Self-Ranking as Religious	−0.04 (−0.13, 0.06)	−0.05 (−0.14, 0.04)	−0.06 (−0.16, 0.04)	0.003 (−0.10, 0.11)	−0.04 (−0.11, 0.04)	−0.02 (−0.11, 0.07)	−0.04 (−0.14, 0.05)	−0.03 (−0.13, 0.08)	−0.03 (−0.12, 0.07)
Observations	270	270	270	270	270	270	270	270	270
R^2^	0.04	0.04	0.04	0.06	0.04	0.04	0.03	0.05	0.03
Adjusted R^2^	0.01	0.01	0.01	0.03	0.02	0.02	0.002	0.02	0.01
Residual Std. Error (df = 261)	0.76	0.78	0.87	0.88	0.63	0.70	0.67	0.87	0.84
F Statistic (df = 8; 261)	1.27	1.38	1.50	1.95	1.53	1.52	1.08	1.62	1.17

*p < 0.05; **p < 0.01; ***p < 0.001.

The only other dimension of religiosity that demonstrated a significant relationship was the frequency of daily spiritual experiences, which showed a protective effect by negatively correlating with Paranoid Ideation (Beta = −0.16). This indicates that more frequent daily spiritual experiences may be linked to lower levels of Paranoid Ideation.

Further analysis assessed the impact of different aspects of religiosity on quality of life. Utilizing the World Health Organization Quality of Life (WHOQOL) score as the outcome measure, the regression model demonstrated a good fit with the data when Overall Quality of Life, Physical Health, and Psychological Health were used as outcomes (Table 5). Interestingly, only the self-perception of being a religious person showed a positive correlation with overall quality of life (Beta = 0.18). For both Physical and Psychological Health, forgiveness was significantly negatively associated with outcomes in physical (Beta = −0.19) and psychological health (Beta = −0.21).

**Table 5:** Associations between religiousness domains on WHOQOL subscales (*n* = 270).

	Overall quality of life	Physical health	Psychological health	Social relations	Environment
	Estimate, (95% CI)	Estimate, (95% CI)	Estimate, (95% CI)	Estimate, (95% CI)	Estimate, (95% CI)
	(1)	(2)	(3)	(4)	(5)
(Constant)	8.13^***^ (6.97, 9.35)	21.99^***^ (19.65, 24.43)	23.15^***^ (20.63, 25.64)	12.18^***^ (10.20, 14.07)	29.44^***^ (25.14, 33.77)
Daily spiritual experiences	−0.02 (−0.08, 0.02)	−0.04 (−0.18, 0.06)	−0.05 (−0.17, 0.05)	−0.003 (−0.10, 0.07)	−0.02 (−0.21, 0.16)
Values	−0.05 (−0.27, 0.14)	0.31 (−0.14, 0.76)	−0.20 (−0.68, 0.24)	−0.26 (−0.60, 0.08)	0.21 (−0.49, 0.87)
Forgiveness	−0.07 (−0.19, 0.06)	−0.30^**^ (−0.51, −0.06)	−0.35^**^ (−0.57, −0.11)	−0.18 (−0.35, 0.001)	−0.49^**^ (−0.84, −0.11)
Private religious practice	−0.04 (−0.09, 0.01)	−0.01 (−0.13, 0.11)	−0.04 (−0.15, 0.07)	−0.01 (−0.09, 0.09)	0.002 (−0.17, 0.18)
Religious coping	−0.02 (−0.08, 0.05)	−0.07 (−0.19, 0.06)	−0.05 (−0.17, 0.09)	0.01 (−0.09, 0.13)	−0.08 (−0.29, 0.14)
Personal religious history	−0.16 (−0.44, 0.12)	−0.14 (−0.64, 0.33)	−0.35 (−0.87, 0.22)	−0.09 (−0.46, 0.28)	−0.01 (−0.90, 0.89)
Organizational religiousness	0.11 (0.0000, 0.21)	0.14 (−0.09, 0.36)	0.26^*^ (0.02, 0.47)	0.004 (−0.18, 0.17)	0.21 (−0.14, 0.54)
Self-ranking as religious	0.23^*^ (0.05, 0.42)	0.37 (−0.10, 0.80)	0.21 (−0.21, 0.64)	0.20 (−0.13, 0.54)	0.41 (−0.27, 1.09)
Observations	270	270	270	270	270
R^2^	0.06	0.06	0.10	0.04	0.04
Adjusted R^2^	0.03	0.03	0.07	0.01	0.01
Residual Std. Error (df = 261)	1.65	3.25	3.38	2.64	5.26
F Statistic (df = 8; 261)	2.03^*^	2.06^*^	3.59^***^	1.44	1.43

*p < 0.05; **p < 0.01; ***p < 0.001.

Contrastingly, no religiousness aspects were significantly linked with Social Relationships domain outcomes. However, the model investigating the influence of religiosity on the Environmental domain was not a good fit, and once again, forgiveness was negatively associated with Environmental quality of life (Beta = −0.20). These results imply that the practice of forgiveness may be associated with reduced satisfaction in physical, psychological, and environmental aspects of life.

The subsequent analysis explored how various dimensions of religiosity influence coping strategies, with findings detailed in Table 1. The regression models generally demonstrated a good fit with the data, except for those examining acceptance, catastrophizing, and blaming others. This analysis revealed a broader range of individual aspects of religiosity associated with different coping techniques compared to the associations observed with previously investigated outcome variables.

Notably, the frequency of daily spiritual experiences had a significant negative association with both refocusing and blaming others (Beta = −0.16 and Beta = −0.19, respectively). Conversely, utilizing religion and spirituality as coping mechanisms was significantly and negatively linked to acceptance (Beta = −0.18), though the presence of zero in the bootstrapped confidence intervals suggests these findings might not be robust.

Forgiveness and the depth of one’s personal religious or spiritual history emerged as the most influential factors. Forgiveness was significantly associated with an increase in self-blame (positively), and decrease in refocusing, planning (both negatively), and putting things into perspective (negatively) (Beta = 0.25, Beta = −0.06, and Beta = −0.16, respectively). In contrast, a deeper personal religious history was positively associated with self-blame, rumination, and refocusing (Beta = 0.22, Beta = 0.13, and Beta = 0.15, respectively).

These patterns indicate that such aspects of religiosity as forgiveness and one’s religious history are primarily linked to maladaptive coping strategies, such as self-blame and rumination, and may negatively affect more adaptive coping strategies, like refocusing.

The final stage of the analysis sought to examine the potential mediating roles of self-blame, positive refocusing, and adopting a new perspective in the relationship between forgiveness and stress. Although personal religious history significantly influenced coping strategies, it was not included in the mediation model due to its lack of association with stress, as previously shown in Table 3.

**Figure 1:**
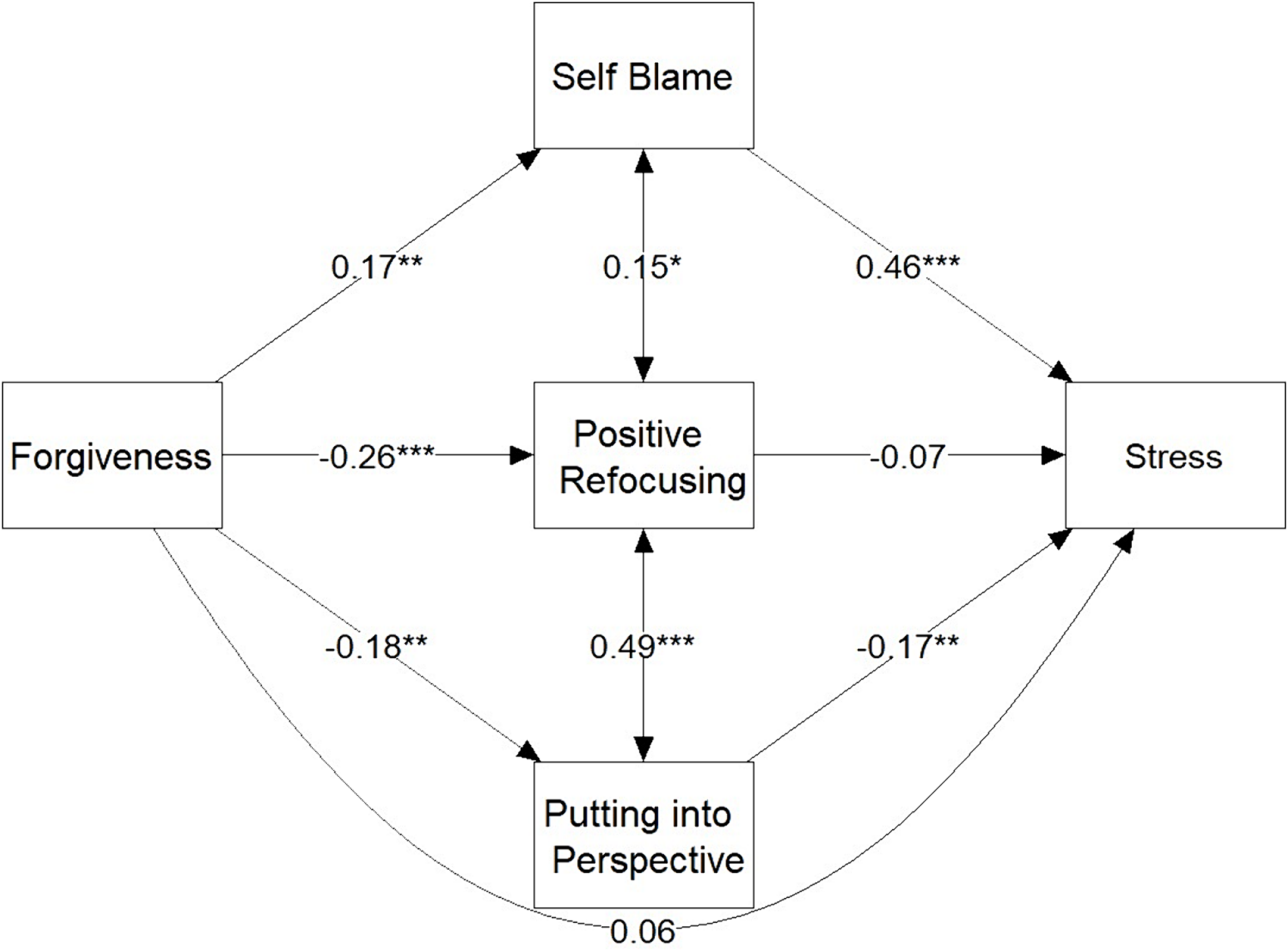
Multiple mediation model.

Initially, a multiple mediation model (Figure 1) was evaluated against a constrained version to assess the equality of indirect effects. The likelihood ratio test, comparing these models, yielded a Chi-squared difference of 3.02 with a *p*-value of 0.22. This result indicates no significant difference between the models, affirming the equality of their indirect effects.

The analysis revealed significant indirect effects for both self-blame (Standardized Estimate = 0.08, *p* = 0.008) and putting things into perspective (Standardized Estimate = 0.03, *p* = 0.045). Although the direct effect of forgiveness on stress was not significant (Standardized Estimate = 0.06, *p* = 0.33), the total effect was significant (Standardized Estimate = 0.18, *p* = 0.002).

## Discussion

In a comprehensive analysis exploring the relationship between various dimensions of religiosity and mental health outcomes, coping strategies, and quality of life among young adults, several key findings emerged. The analysis revealed that forgiveness, a central aspect of some religious doctrines, was significantly associated with mental health outcomes. Conversely, self-perception as a religious person and daily spiritual experiences demonstrated positive effects on quality of life and certain coping mechanisms. Notably, mediation models highlighted how forgiveness can result in stress by leading to maladaptive or undermining adaptive coping strategies. Specifically, it increases self-blame but decreases the ability to put things into perspective. These results emphasize the nuanced and multifaceted impact of religiosity on mental health and coping, suggesting that while certain aspects of religiosity can enhance personal wellbeing, some, especially forgiveness, may exacerbate negative mental health outcomes and maladaptive coping strategies.

Forgiveness is often considered an important component of various religious doctrines, including Christianity, Islam, and Buddhism. In Christianity, for example, forgiveness is a key aspect of Jesus’ teachings, although it is not the sole central aspect of the entire doctrine. In Islam, forgiveness is emphasized as a virtue and an act that brings the believer closer to God. In Buddhism, forgiveness is associated with the path to liberation from negative emotions and karmic consequences.

In our study, we worked with a sample that included individuals from various religious backgrounds, including the aforementioned doctrines. This allowed us to examine the impact of forgiveness within different religious contexts. Our findings confirm the duality of the impact of religion and spirituality on mental health (Koenig et al., 2012; Papaleontiou-Louca, 2021; Fradelos et al., 2020).

### Implications of findings

The implications of our findings are significant for both clinical practice and future research. Clinicians should be aware of the potential negative impacts of forgiveness on mental health, particularly in contexts where individuals may be encouraged to forgive as part of their religious practice. Therapeutic interventions should consider the individual’s religious background and how it influences their coping strategies. Furthermore, our findings highlight the need for a more personalized approach in mental health care that takes into account the complex interplay between religiosity, spirituality, and mental health.

### Limitations

There are several limitations to our study that must be acknowledged. First, the use of self-reported measures for assessing R/S and mental health outcomes can introduce bias. Participants may respond in socially desirable ways or may have difficulties accurately recalling or evaluating their experiences and behaviors. Second, our study does not track changes in R/S and mental health outcomes over time. Longitudinal data would provide more insights into how these relationships evolve and whether changes in R/S practices impact mental health trajectories. Additionally, our sample, while diverse, may not be representative of all religious or spiritual contexts, limiting the generalizability of our findings.

### Future directions and considerations

Given the complex and multifaceted nature of religiosity and spirituality (R/S) on mental health, future research should consider the following:

*Longitudinal studies*: Conduct longitudinal studies to better understand the causal relationships between R/S and mental health outcomes over time. Cross-sectional designs, like the one used in this study, provide valuable snapshots but cannot definitively establish causality.

*Diverse populations*: Include more diverse populations in terms of religious affiliation, cultural background, and geographic location. This will help generalize findings and uncover unique interactions between R/S and mental health across different groups.

*Mechanisms of impact*: Further investigate the mechanisms through which R/S influences mental health. For instance, while forgiveness can have both positive and negative impacts, understanding the conditions under which it leads to maladaptive coping versus adaptive coping can inform targeted interventions.

*Contextual factors*: Examine contextual factors such as community support, religious involvement, and the broader social and cultural environment that may moderate the relationship between R/S and mental health.

*Interdisciplinary approaches*: Utilize interdisciplinary approaches combining psychology, sociology, theology, and public health to provide a more comprehensive understanding of the role of R/S in mental health.

By addressing these areas, future research can build on the findings of this study and contribute to a more nuanced understanding of how religiosity and spirituality interact with mental health, ultimately informing better clinical practices and supportive interventions for individuals seeking to integrate their spiritual beliefs with their mental health care.

This study provides new insights into the existing research on religion and spirituality (R/S) and their impact on mental health by focusing on several key areas that have not been sufficiently explored. One of the main theoretical contributions is the emphasis on the duality of the impact of religion and spirituality on mental health, particularly in the context of forgiveness, which can have both positive and negative effects on psychological well-being.

### Duality of forgiveness

Our study identified that forgiveness, as a central aspect of some religious doctrines, can lead to increased stress and maladaptive coping strategies, such as self-blame and a reduced ability to see things in perspective. This finding provides a new perspective on the theoretical understanding of how religious practices, traditionally seen as positive, can have complex and sometimes harmful effects on mental health.

### Conceptual framework of R/S

The study contributes to the expansion of the conceptual framework for examining R/S by exploring various dimensions of religion and spirituality (e.g., daily spiritual experiences, personal religious history) and their specific influences on different aspects of mental health and quality of life. This allows for a better understanding of how different aspects of R/S interact with psychological processes and how they can be integrated into clinical practices.

### Mechanisms of influence

Our findings highlight the need for further investigation into the mechanisms through which R/S influence mental health. Identifying that forgiveness can lead to maladaptive coping strategies raises new questions about the conditions under which these religious practices lead to positive versus negative outcomes.

### Individual and contextual factors

Emphasizing the individuality of the impact of R/S on mental health provides an important theoretical contribution by pointing out how specific religious beliefs, personal experiences, and context can modify this relationship. This underscores the significance of a personalized approach in therapeutic interventions aimed at integrating spiritual and religious beliefs.

In this way, this study not only expands theoretical knowledge in the area of R/S and mental health but also provides practical implications for clinical practice and future research.

## Conclusion

The finding that forgiveness may be a risk factor associated with regularly elevated depressive symptoms, stress, and maladaptive coping strategies such as self-blame and ruminating over problems, while negatively affecting physical, psychological, and environmental quality of life, clearly points to the need to examine the inner aspects of individual religions and spiritualties. These findings suggest that religious and spiritual beliefs may play a key role in how people experience and manage the emotional burdens and difficulties of life.

Studying these inner parameters of religion and spirituality can help to better understand the mechanisms behind how these aspects of life affect the mental and physical health of individuals. This could lead to better use of religious and spiritual resources as a means to protect and enhance mental health and manage stress. It could also provide suggestions for the development of interventions aimed at promoting forgiveness and healthier ways of coping that could bring about significant improvements in the wellbeing of individuals, families and communities.

At the same time, it is important to consider the context and particularities of individual religions and spiritual traditions, as their influence on individual experiences of forgiveness and stress management may vary. Research in this direction could contribute to understanding these differences and to developing personalized approaches to promoting mental health and wellbeing. Overall, exploring the inner parameters of religion and spirituality opens the door to a deeper understanding of the human experience and to finding new ways to improve quality of life.

## Data Availability

The raw data supporting the conclusions of this article will be made available by the authors, without undue reservation.
